# Frailty Confers High Mortality Risk across Different Populations: Evidence from an Overview of Systematic Reviews and Meta-Analyses

**DOI:** 10.3390/geriatrics5010017

**Published:** 2020-03-12

**Authors:** Richard Ofori-Asenso, Ken Lee Chin, Berhe W. Sahle, Mohsen Mazidi, Andrew R. Zullo, Danny Liew

**Affiliations:** 1 Department of Epidemiology and Preventive Medicine, Monash University, Melbourne, VIC 3181, Australia; ken.chin@monash.edu (K.L.C.); danny.liew@monash.edu (D.L.); 2 Department of Pharmacy, Faculty of Health and Medical Sciences, University of Copenhagen, 2 2300 Universitetsparken, Copenhagen, Denmark; 3 Melbourne Medical School, University of Melbourne, Parkville, VIC 3010, Australia; 4 Melbourne School of Population and Global Health, University of Melbourne, Parkville, VIC 3053, Australia; berhe.sahle1@gmail.com; 5 Twin Research and Genetic Epidemiology, Kings College London, London SE1 7EH, UK; mazidi_ns@yahoo.com; 6 Department of Health Services, Policy, and Practice, Brown University School of Public Health, Providence, Rhode Island, RI 02912, USA; andrew_zullo@brown.edu

**Keywords:** frailty, mortality, evidence synthesis, meta-analyses, umbrella review

## Abstract

We performed an overview of systematic reviews and meta-analyses to summarize available data regarding the association between frailty and all-cause mortality. Medline, Embase, CINAHL, Web of Science, PsycINFO, and AMED (Allied and Complementary Medicine) databases were searched until February 2020 for meta-analyses examining the association between frailty and all-cause mortality. The AMSTAR2 checklist was used to evaluate methodological quality. Frailty exposure and the risk of all-cause mortality (hazard ratio [HR] or relative risk [RR]) were displayed in forest plots. We included 25 meta-analyses that pooled data from between 3 and 20 studies. The number of participants included in these meta-analyses ranged between <2000 and >500,000. Overall, 56%, 32%, and 12% of studies were rated as of moderate, low, and critically low quality, respectively. Frailty was associated with increased risk of all-cause mortality in 24/24 studies where the HR/RRs ranged from 1.35 [95% confidence interval (CI) 1.05–1.74] (patients with diabetes) to 7.95 [95% CI 4.88–12.96] (hospitalized patients). The median HR/RR across different meta-analyses was 1.98 (interquartile range 1.65–2.67). Pre-frailty was associated with a significantly increased risk of all-cause mortality in 7/7 studies with the HR/RR ranging from 1.09 to 3.65 (median 1.51, IQR 1.38–1.73). These data suggest that interventions to prevent frailty and pre-frailty are needed.

## 1. Introduction

Frailty, defined as a geriatric syndrome characterised by increased vulnerability to even minor stressors and decreased physiological reserve [1,2], is common among older people. Globally, it has been estimated that about 4.3% of older adults aged 60 years and older will develop frailty per year [3]. However, the prevalence of frailty varies widely among older people: 4.0–59.1% in community-dwelling older adults [4], 10.4–37.0% in general surgery patients [5], 6.0–86.0% in cancer patients [6], and 19.0–75.6% in nursing home residents [7].

Numerous studies have shown frailty to be associated with many negative health outcomes including increased risk of hospitalizations [8], disability [9], falls [10], fractures [11], delirium [12], and institutionalization [13]. Owing to these adverse effects, frailty is associated with higher healthcare use and cost burden [14,15,16].

Moreover, frailty has been associated with increased risk of mortality and premature death [1,17,18], and this effect has been suggested to be stable across different generational cohorts [19]. In particular, Shamliyan and colleagues have suggested that 3–5% of deaths among community-dwelling adults could be averted if there are concerted efforts to prevent frailty [20]. The exact mechanisms underpinning increased mortality among people who are frail remains unclear, although, there are suggestions that reduced physical and cognitive functions associated with frailty leads to poor prognosis [21,22]. Others have also indicated that it is the underlying clinical conditions associated with frailty that are ultimately the cause of death [23,24]. For example, an underlying physical or even psychological condition can give rise to weight loss, which contributes to the development of frailty [25]. Whether or not the effects of frailty on mortality risk are ubiquitous or more profound in selected subgroups is also unclear.

Thus, this umbrella review summarizes evidence generated from systematic reviews and meta-analyses that examined the association between frailty and all-cause mortality across different population groups and settings.

## 2. Methods

This review was performed according to the recommendations outlined in the Meta-analysis of Observational Studies in Epidemiology [26], the Preferred Reporting Items for Systematic Reviews and Meta-Analyses statements [27] (Supplementary Table S1), and the Cochrane Collaboration Handbook [28].

### 2.1. Search Strategy and Study Selection

A systematic literature search of Medline, Embase, CINAHL, Web of Science, PsycINFO, and AMED (allied and complementary medicine) databases for systematic reviews and meta-analyses examining the association between frailty and the risk of all-cause mortality. The search was first performed in August 2019 and was last updated in February 2020. The main keywords used included “frailty OR frailty syndrome OR frail elderly OR geriatric syndrome OR geriatric disorder” AND “systematic review OR meta-analysis OR review”. The full search strategy is presented in Supplementary Table S2. In addition, the reference lists of included systematic reviews and meta-analyses were manually screened. The literature search was independently performed by two reviewers (RO and KLC), and disagreements were resolved by consensus involving a third author (BWS).

### 2.2. Selection Criteria

The inclusion criteria were as follows: (1) participants: any population; (2) study design: meta-analyses that reported pooled risk estimates (hazard ratios [HRs] or risk ratios [RRs]; (3) exposure: frailty or pre-frailty as defined by any criteria; and (4) outcome: risk of all-cause mortality. We restricted the review to only studies reporting HRs and RRs as these are measures are largely comparable, and in other reviews have been pooled together [28]. The exclusion criteria were as follows: (1) studies published in languages other than English; (2) meta-analyses that did not follow the methodology of a systematic review; (3) meta-analyses that pooled frailty scores without categorisation; and (4) systematic reviews without meta-analysis.

### 2.3. Data Extraction

Two researchers (KLC and BWS) independently extracted the following data from the original studies using a standardized data collection form: (1) author identification and year of publication; (2) databases searched for systematic review; (3) search date; (*4*) number of studies included; (5) age of the participants; (6) length of follow-up; (7) number of frail and non-frail populations; (8) frailty assessment tool used; (9) reported risk estimates; (8) heterogeneity reported; and (9) AMSTAR2 risk of bias rating [29]. We also extracted age- or sex-specific risk estimates if reported. Where studies reported effect of frailty at different follow-up periods, we prioritized the data on long-term mortality effects. Disagreements in data collection were resolved by discussion involving a third author (RO).

### 2.4. Quality Assessment

Two investigators (BS and KLC) independently assessed the methodological quality of each study after concealment of information about the authors, affiliations, date and source. A standardized checklist, the AMSTAR2 appraisal tool, for reporting systematic reviews and meta-analyses was used [29]. This checklist includes 16 criteria, each referring to a relevant methodological aspect of the study. For each review, we applied a rating of high, moderate, low, or critically low for the overall confidence in the results. The ratings were performed by two reviewers (BS and KLC) and any disagreements were resolved via consensus involving third reviewer (RO).

### 2.5. Data Synthesis

To depict the relationship between frailty and all-cause mortality, the risk estimates (HR/RR) and corresponding 95% confidence intervals (CIs) for each meta-analysis were displayed in a forest plot [30,31,32]. Using the point HR/RR from each study, we also calculated the median risk estimate along with corresponding interquartile range (IQR) to describe the distribution of the reported effect of frailty on all-cause mortality. The level of heterogeneity across individual meta-analysis was assessed via the *I*^2^ statistic, and depending on its values heterogeneity was considered as the following: might not be important (0–40%), may represent moderate heterogeneity (30–60%), substantial heterogeneity (50–90%), or considerable heterogeneity (75–100%) [28]. Forest plots were produced using StataSE software version 16 (StataCorp, TX, USA), which displayed data from original meta-analyses without including any additional analyses.

## 3. Results

### 3.1. Characteristics of Included Studies

**Figure 1:**
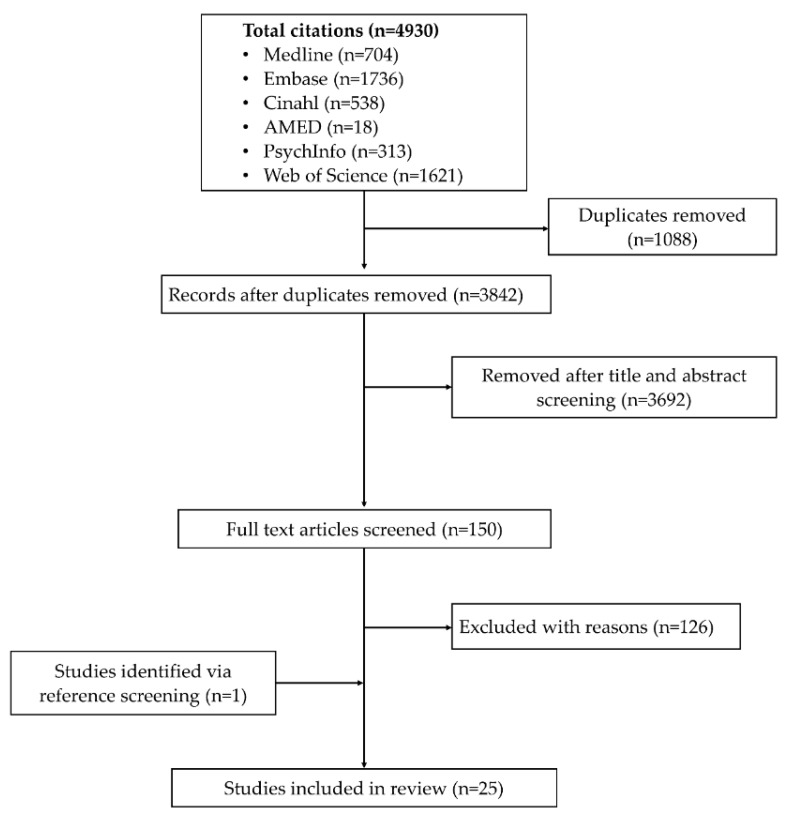
Flow chart of studies’ selection process.

Our search identified 4930 citations. A total of 150 articles were selected for full text examination while others were removed following initial screening based on titles and abstract or duplicates. Of the 150 articles, 24 were eligible for analysis. One additional study was retrieved via reference screening. The studies’ selection process is summarised in Figure 1.

The descriptive characteristics of the included studies are presented in Table 1. The meta-analyses were published between 2015 and 2019 and included between three and 24 studies with sample sizes ranging from <2000 to >500,000 participants. All the systematic reviews and meta-analyses conducted their search in two or more databases. The meta-analyses focused on community-dwelling older adults (*n* = 3); patients who had undergone transcatheter aortic valve implantation or replacement (*n* = 3), percutaneous coronary intervention (PCI; *n* = 1), or left ventricular assist device implantation (*n* = 1); vascular surgery patients (*n* = 1); patients with chronic kidney disease or end-stage renal disease (*n* = 1); patients with heart failure (*n* = 4), diabetes (*n* = 1), acute coronary syndrome (ACS; *n* = 3), or multiple myeloma (*n* = 1); patients who had been admitted to an intensive care unit (ICU; *n* = 1), nursing home residents (*n* = 1); patients who had undergone a range of other surgical interventions (*n* = 3) or those hospitalized for a range of medical conditions (*n* = 1).

The heterogeneity (*I*^2^) across the individual meta-analyses was variable and ranged from 0% to 100%. Fifty-six percent (14/25) of studies reported substantial to a considerable level of heterogeneity. In the studies which reported frailty prevalence, the baseline prevalence of frailty ranged from 7.9% to 86.6%.

After evaluation of the risk of bias by the AMSTAR 2 tool, 56% (14/25), 32% (8/25), and 12% (3/25) were rated to be of moderate, low, and critically low quality, respectively. The critical domains that were most frequently lacking were justification for exclusion of individual studies (22 out of 25), lack of protocol being registered before review commencement (11 out of 25) and assessment of present and likely impact of publication bias (eight out of 25). The breakdown of the AMSTAR 2 assessment for each study is presented in Supplementary Table S3.

### 3.2. Association Between Frailty and All-Cause Mortality

**Figure 2:**
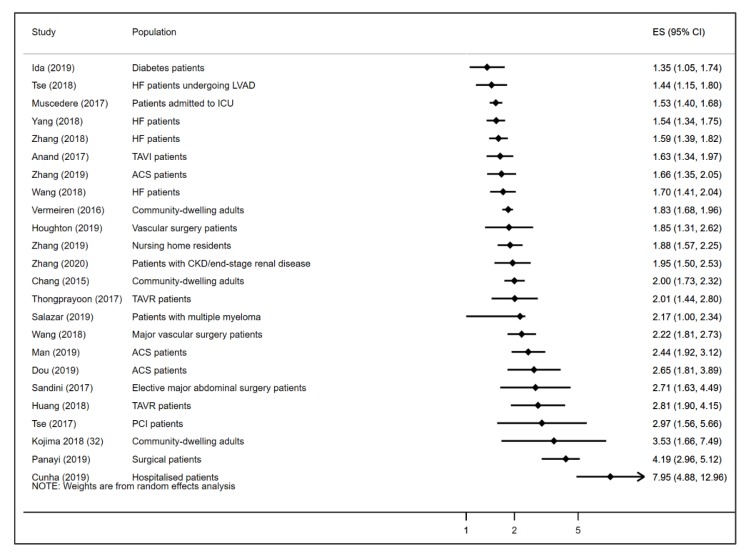
Forest plot showing the reported HR/RR of the association between frailty and all-cause mortality across the included meta-analyses. HF = heart failure; ACS = acute coronary syndrome; PCI = percutaneous coronary intervention; TAVI = TAVI = transcatheter aortic valve implantation patients; TAVR = transcatheter aortic valve replacement, LVAD = left ventricular assist device implantation; ICU = intensive care unit.

A total of 24 meta-analyses reported the association between frailty and the risk of all-cause mortality. All 24 studies found that frailty was associated with increased risk of all-cause death. The reported HR/RRs of the effect of frailty on all-cause mortality ranged from 1.35 to 7.95. The lowest HR/RR was reported among diabetes patients and the highest among hospitalized patients. The median HR/RR estimate of frailty on all-cause mortality across the included studies was 1.98 (IQR 1.65–2.67). Figure 2 displays a forest plot of the of the reported HR/RR of the association between frailty and all-cause mortality in the included studies. None of the studies reported age- or sex-specific pooled data.

### 3.3. Association between Pre-Frailty and All-Cause Mortality

**Figure 3:**
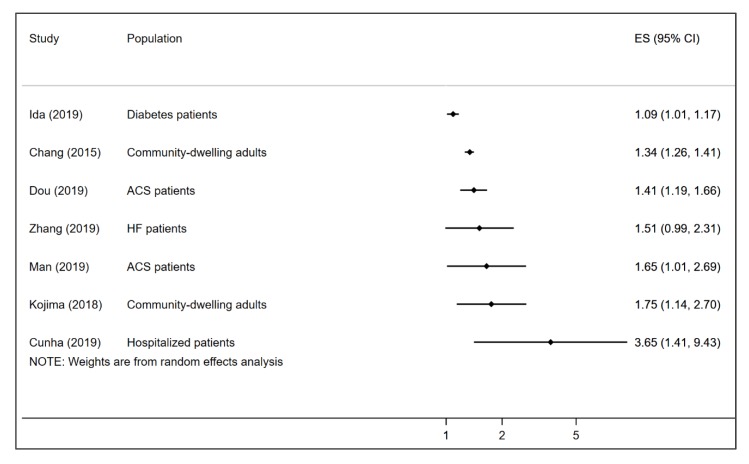
Forest plot showing the reported HR/RR of the association between pre-frailty and all-cause mortality across the included meta-analyses. ACS = acute coronary syndrome; HF = heart failure.

All seven meta-analyses that reported the association between pre-frailty and all-cause mortality found that pre-frailty was associated with increased risk of all-cause mortality. The reported HR/RRs of the effect of pre-frailty on all-cause mortality ranged from 1.09 among diabetes patients to 3.65 among hospitalized patients. The median HR/RR estimate of frailty on all-cause mortality across the included studies was 1.51 (IQR 1.38–1.73). Figure 3 displays a forest plot of the association between pre-frailty and all-cause mortality in the included studies. None of the studies reported age- or sex-specific pooled data.

## 4. Discussion

Increasingly, frailty has been recognized as a marker of poor ageing [58], and this syndrome is associated with many negative health outcomes [9,10,11,12,15]. However, the absence of a lack of a universally accepted definition creates clinical and research conundrum [59,60]. Moreover, while frailty is expressed over a broad spectrum of severity, defining the point at which frailty begins is challenging [1]. An equally important issue also relates to identification of the tipping point at which the progression of frailty begins to exert negative effects on survival [20].

With the above in mind, this overview of systematic reviews and meta-analyses provides a synthesis of the current available evidence of the association between frailty and the risk of all-cause mortality. We found consistent data across different populations and settings suggesting that both frailty and pre-frailty are associated with increased risk of all-cause mortality. Across the included studies, frailty was on average associated with a two-fold increased risk of death, although the risk of mortality associated with pre-frailty appeared to be much lower.

Although all the included studies suggested frailty and pre-frailty to be associated with increased risk of all-cause mortality, there was significant heterogeneity in the effect sizes. For example, while one study reported frailty to be associated with about two times increased risk of death in community-dwelling adults [42], another study reported frailty to be associated with a nearly eight-fold increase in risk of mortality among patients hospitalized for a range of conditions [56]. These data suggest that the mortality effect of frailty across different populations may be graded and could also be related to other underlying pathological risk factors/stressors that exacerbate the impact of frailty to varied extents in different populations [21,22].

As frailty is associated with increased risk of mortality, screening for frailty may be useful for all older adults. However, frailty screening remains a highly debated issue especially in relation to screening eligibility, as well as where and when it should be performed [3,61]. Regardless, within specialized geriatric ambulatory care settings and long-term care facilities, the use of comprehensive geriatric assessments that incorporate the deficit accumulation definition of frailty may be more feasible to screen for frailty [62,63], whereas the frailty phenotype may be easier adopt within primary care settings to identify frail individuals [20].

Healthcare providers need to recognize frailty both as an important baseline condition and an outcome among older adults. To this end, improved understanding of the risk factors of frailty is important. Several factors are associated with increased risk of frailty including individual chronic morbidities such as diabetes, cancers, or depression or their co-existence [3,64,65]. Psychosocial (e.g., social isolation) and lifestyle factors also play important roles in the development of frailty [3,66,67]. Regardless, studies also suggest that both frailty and pre-frailty can be reversed [68,69]. Thus, to minimize the risk of frailty development, delay its progression or promote reversal, multidisciplinary interventions that aim to improve physical, cognitive, and social functioning are required [20,70,71,72]. In the context of primary care settings, relative effectiveness and ease of implementation of programs at preventing or slowing frailty progression, ranked from highest to lowest, are strength training, protein supplementation, comprehensive geriatric assessments, home visits and behavioral change interventions [72].

Although the present overview provides important insights, some limitations should be considered. First, the criteria for ascertaining death varied across the primary studies of the meta-analyses, and therefore misclassification may have affected the estimates of the association between frailty and the risk of mortality. Secondly, some primary studies may have been included in more than one meta-analysis and thus the influence of these studies would have been inflated. Thirdly, some meta-analyses involved primary studies that showed substantial heterogeneity, which may limit the validity of their, and hence our, conclusions. Furthermore, although the prevalence of frailty and its mortality effects could vary by age, sex or other characteristics [73,74,75], we could not examine this in detail due to limited data. We could also not examine the presence of publication bias due to the design of our study and the methodology used. Moreover, some meta-analyses were based on small number of studies showed large effect sizes, which may be suggestive of publication bias in reviews. Lastly, further bias may have arisen from our limitation of component studies to English, although the extent of this bias, if present, would likely have been small.

## 5. Conclusions

Across different populations and settings, frailty defined using multiple criteria is associated with two-fold increased risk of all-cause mortality. Thus, public health interventions to prevent or slow the progression of frailty may prevent avoidable deaths.

## Figures and Tables

**Table 1:** Descriptive characteristics of the included meta-analyses.

Author	Databases Searched	Search Period	No. of Studies Pooled	Population/Setting	Age	% Women	Countries of Included Studies	Follow up Duration	Total Sample (% Frail)	Exposures Assessed	Pre-Defined Frailty Measurement Criteria	Frailty Mortality HR/RRs (95% CI)	*Model used*	*I* ^2^	AMSTAR Quality Rating
Anand et al. [33]	Medline, Embase and Cinahl	1 January 2000 to 1 June 2015	7	TAVI patients	Mean age: 81–86 y	48–66%	Switzerland, Canada, Germany, Netherlands, Italy, USA	>30 days	3159 (23.2%)	Frailty	Any	1.63 (1.34–1.97) ^a^	RE	66.0%	Moderate
Huang et al. [34]	PubMed, Embase and Cochrane library	Inception to January 2018	10	TAVR patients	NR	NR	USA, Poland, Germany, Spain, Japan, Switzerland	>6 months	2992 (32.5%)	Frailty	Any	2.81 (1.90–4.15) ^b^	RE	84.0%	Moderate
Ida et al. [35]	Medline, Cochrane central and Clinicaltrials.gov	Inception to 1 December 2018	4 (Frail vs. non-frail) 2 (pre-frail vs. non-frail)	Patients with diabetes	Mean age: 56–76 y	46–67%	Italy, USA, Spain, Taiwan	3–12 y	563,761 (NR)	Frailty, Pre-frailty	Any	Frailty: 1.35 (1.05–1.74) ^a^ Pre-frailty: 1.09 (1.01–1.17) ^a^	RE	92.0% (Frailty) 89.0% (Pre-frailty)	Moderate
Kojima et al. [36]	Embase, Scopus, Medline, Cinahl PsycINFO and Google scholar	Inception to March 2018	3	Community-dwelling middle-aged and older adults	Mean age: ≥59.9 y	0–53.4%	UK, Mexico, Italy, Belgium, Denmark, France, Greece, Italy, Netherlands, Spain, Sweden, Switzerland	2.4–4.3 y	9273 (NR)	Frailty, Pre-frailty	FRAIL scale	Frailty: 3.53 (1.66–7.49) ^a**^ Pre-frailty: 1.75 (1.14–2.70) ^a^	RE	79.0% (Frailty) 64.0% (Pre-frailty)	Moderate
Man et al. [37]	PubMed and Embase	Inception to October 1 2018	5 (Frailty) 3 (Pre-frailty)	Patients with ACS	≥65 y	32.8–48.9	Canada, Spain, France, Sweden	≥12 months	1270 (38.4%)	Frailty, Pre-frailty	Any	Frailty: 2.44 (1.92–3.12) ^a^ Pre-frailty:1.65 (1.01–2.69) ^a^	FE	0.0% (Frailty), 0.0% (pre-frailty)	Critically low
Muscedere et al. [38]	Cochrane central, Medline, Embase, PubMed, Cinahl and Clinicaltrials.gov	Inception to April 2017	6	Patients admitted to the ICU	Mean age: 57.1–84.0 y	NR	USA, Canada, Turkey, France	>6 months	2484 (42.8%)	Frailty	Any	1.53 (1.40–1.68) ^b^	RE	0.0%	Moderate
Panayi et al. [39]	PubMed and Cochrane central	Inception to 1 January 2018	11	Surgical patients (All surgeries)	NR	NR	USA, Canada	NR	523,598 (70.7%)	Frailty	Modified frailty index (mFI)	4.19 (2.96–5.12) ^b^	RE	91.0%	Moderate
Salazar et al. [40]	Medline, Embase, Scopus, Cochrane Database of Systematic Reviews, Cochrane Central, and Clinicaltrials.gov	Inception to August 2018	3	Patients with multiple myeloma	Median age: 58–74	NR	Italy, Czech Republic, Netherlands, China, Germany	13–28 months	1622 (NR)	Frailty	Any	2.17 (1.00–2.34) ^a^	RE	33.7%	Critically low
Sandini et al. [41]	Medline, Embase, PubMed, Cochrane and Scopus libraries	1990 to January 2017	8	Elective major abdominal surgery patients	NR	NR	USA, Korea, Norway, Netherlands	Up to 1 y	16,825 (NR)	Frailty	Any	2.71 (1.63–4.49) ^a^	RE	88.3%	Moderate
Shu-Fang et al. [42]	Medline and CINAHL databases and the Cochrane Library	January 2001 to July 2014	11	Community-dwelling older adults	≥65 y	NR	France, Spain, USA, Israel, Finland	1.5–10 y	35538 (NR)	Frailty, Pre-frailty	Fried (CHS) criteria	Frailty:2.00 (1.73–2.32) ^a^ Pre-frailty: 1.34 (1.26–1.41) ^a^	RE	61.7% (Frailty) 0.0% (Pre-frailty)	Moderate
Thongprayoon et al. [43]	Medline, Embase, the Cochrane Central Register of Controlled Trials, and the Cochrane Database of Systematic Reviews	Inception to November 2016	8	TAVR patients	NR	NR	USA, Germany, France	NR	10,498 (NR)	Frailty	Any	2.01 (1.44–2.80) ^b^	RE	58.0%	Moderate
Tse et al. [44]	PubMed and EMBASE	Inception to July 23 2017	8	Patients who have undergone PCI	Mean age 69 y	32	Spain, Indonesia, UK, Japan, USA	Mean 2.5 y	2332 (NR)	Frailty	Any	2.97 (1.56–5.66) ^a^	RE	79.0%	Low
Tse et al. [45]	PubMed and Embase	Inception to September 11, 2017	7	Advanced Heart Failure Patients Undergoing Left Ventricular Assist Device Implantation	Mean age 48–69 y	20.8		Mean 13 months	2942 (13.5%)	Frailty	Any	1.44 (1.15–1.80) ^a^	FE	0.0%	Moderate
Vermeiren et al. [46]	PubMed, Web of Knowledge and PsycINFO	Inception to January 2016	17	Community-dwelling older adults	≥65 y	NR	USA, France, Spain, Cuba, Dominican Republic, Venezuela, Mexico, Peru, India, China, Italy, Finland	10–120 months	150,763 (NR)	Frailty	Any	1.83 (1.68–1.98) ^a/b^	n.s	98.0%	Low
Wang et al. [47]	Medline, Embase, Cochrane, Scopus	Inception to September 2017	9	Patients who have undergone major vascular surgery	Mean age ≥59.1 y	NR	Canada, USA, Japan	1–8.4 y	1957 (35.5%)	Frailty	Any	2.22 (1.81–2.73) ^a^	RE	0.0%	Moderate
Wang et al. [48]	PubMed, Embase, Cochrane, Web of science	Inception to November 8 2017	6	Patients with heart failure	Mean age: 82.6 y	17.6	USA, Italy, Spain	>30 days	1747 (53.0%)	Frailty	Any	1.70 (1.41–2.04) ^a^	FE	0.0%	Low
Yang et al. [49]	Medline, Embase, Cochrane Central	January 1966 to March 2018	8	Patients with chronic heart failure	Mean age: 73.4 y	40.1	USA, Italy, Australia, Spain	NR	2645 (50.7%)	Frailty	Any	1.54 (1.34–1.75) ^a^	RE	0.0%	Moderate
Zhang et al. [50]	Medline, Embase, Cochrane Central	Inception to October 2018	14	Nursing home residents	Mean age:84.9	72.1	France, Spain, Canada, Belgium, USA, Japan, Poland, China, Australia	0.5–9 y	9076 (53.9%)	Frailty	Any	1.88 (1.57–2.25) ^a^	RE	47.8%	Moderate
Zhang et al. [51]	Medline, Embase, Cochrane Central	Inception to October 2018	3	Heart failure patients	NR	NR	USA, Spain	NR	NR	Frailty	Any	Pre-frailty: 1.51 (0.99–2.31) ^a^	RE	0.0%	Critically low
Zhang et al. [52]	PubMed and Embase	Inception to December 3 2017	20	Patients with heart failure	Mean age: 79.9 y	11.1–60.9	USA, Spain, Italy, Australia, Canada, UK, Hong Kong	0.5–12 y	17201 (9.2–76%)	Frailty	Any	1.59 (1.39–1.82) ^a^	RE	55.0%	Low
Dou et al. [53]	PubMed and Embase	Inception to July 1 2018	7	Patients with ACS	≥65 y	NR	Spain, France, Sweden, Canada, Japan, South Africa, Scandinavia, India, East Asia, Australia, New Zealand	In hospital to 56.4 months	6658 (12.3%)	Frailty	Any	Frailty: 2.65 (1.81–3.89) ^a^ Pre-frailty: 1.41 (1.19–1.66) ^a^	RE	60.2% (Frailty) 0.0% (Pre-frailty)	Low
Zhang et al. [54]	PubMed, Web of Science, Embase and Cochrane Central databases	Inception to June 2019	11	Patients with CKD or end-stage renal disease	Mean age: >45.0 y		USA, South Korea, India	1–17 y	127037 (7.9–82.0%)	Frailty	Fried (CHS)	1.95 (1.50–2.53) ^a^	RE	82.0%	Low
Houghton et al. [55]	Cinahl, PsycInfo, and Scopus	inception to September, 2018	9	Vascular surgery patients	NR		USA, Japan, UK	NR	2904	Frailty	Any	1.85 (1.31–2.62) ^a^	RE	74.0%	Low
Cunha et al. [56]	PubMed, Embase, Web of Science, Lilacs, Cinahl, PsycInfo and Google Scholar	Inception to March 2019	4 (Frailty); 3 (Pre-frailty)	Hospitalized patients	NR		NR	7–12 months	2119 (86.6%)	Frailty, Pre-frailty	Any	Frailty: 7.95 (4.88–12.96)^a^, Pre-frailty: 3.65 (1.41–9.43) ^a^	RE	0.0% (Frailty), 28.0% (pre-frailty)	Moderate
Zhang et al. [57]	Medline, Embase, and Cochrane Central	Inception to December 2018		Patients with ACS	NR		NR	15–60 Months	4665 (12.8%)	Frailty	Any	1.66 (1.35–2.05) ^a^	FE	11.0%	Low

a = hazard ratio, b = risk ratio, HF = heart failure, ACS = acute coronary syndrome, CI = confidence interval, CKD = chronic kidney disease, FE = fixed effect model, ICU = intensive care unit; n.s. = not stated, RE = random effect model, TAVI = transcatheter aortic valve implantation patients; TAVR = transcatheter aortic valve replacement, LVAD = left ventricular assist device implantation;** in this study the reference group was only robust people.

## Supplementary Materials

The following are available online at https://www.mdpi.com/2308-3417/5/1/17/s1, Table S1: PRISMA Checklist; Table S2: Search strategy for Ovid Medline which was adapted to other databases; Table S3: AMSTAR2 grading for each study.
